# Tuberculosis

**Published:** 1903-07

**Authors:** 


### Books and Magazines.

---

Tuberculosis—Recast from Lectures Delivered at Rush Medical College, in affiliation with the University of Chicago. By Norman Bridge, A. M., M. D., Emeritus Professor of Medicine in Rush Medical College; Member of the Association of American Physicians. Handsome 12mo volume of 302 pages, illustrated. Philadelphia, New York and London: W. B. Saunders & Company. 1903. Cloth, $1.50 net.

This work represents the substance of the lectures on "Medical

Tuberculosis" delivered by Professor Bridge at Rush Medical College during the past three years. It is essentially practical in character, containing as it does a concise and at the same time complete description of the best known means, both preventive and curative, of treating the disease. The author in his preface very aptly says: "In this day of better hope for the victims of this amazing disease, and better knowledge of how to prevent it, a new science and a new gospel need to be taught, to the end that both the profession and the public may be aroused to their duty and opportunities."

The book is one that every general practitioner can and should read. R. and L.

